# Reconsidering the role of L2 self-motivational and affective factors in AI-mediated informal digital learning of English: a mixed-methods study

**DOI:** 10.3389/fpsyg.2026.1696171

**Published:** 2026-01-29

**Authors:** Haomin Tommy Li, Ping Yan

**Affiliations:** 1 Faculty of Humanities and Social Sciences, City University of Macau, Macao, Macao SAR, China; 2 School of Foreign Languages, China West Normal University, Nanchong, China

**Keywords:** anxiety, artificial intelligence-mediated informal digital learning of English, enjoyment, self-efficacy beliefs, motivation, positive psychology

## Abstract

**Introduction:**

Growing scholarly interest has emphasized the significance of non-cognitive variables in influencing learners’ informal English acquisition through artificial intelligence (AI)-enhanced digital platforms. Despite this, research remains sparse on how motivational constructs, emotional responses, particularly enjoyment, and anxiety resulting from AI-assisted learning (AAL), and participation in AI-mediated informal digital English learning (AI-IDLE) collectively contribute to learners’ self-efficacy beliefs in speaking English as a foreign language (EFL).

**Methods:**

Employing an explanatory sequential mixed-method design, this study surveyed 308 EFL secondary students in China, followed by in-depth interviews with eight voluntary respondents. Quantitative data were analyzed using structural equation modeling, while qualitative data underwent thematic analysis involving both open and axial coding procedures.

**Results:**

Path results demonstrated that AAL negatively predicted EFL learners’ AI-IDLE engagement. However, AI-IDLE exerted no statistically significant influence on students’ self-reported self-efficacy in spoken English. Notably, enjoyment emerged as a mediator in the paths from the ideal second language (L2) self to both AI-IDLE and self-efficacy beliefs in speaking capacity. Qualitative data based on narrative descriptions evinced the heterogeneity of motivational and emotional factors of students operating in AI-supported informal learning situations.

**Discussion:**

Through a combination of quantitative results with interpretative reflections, this study presents some useful guidelines for maximizing student participation with respect to AI-IDLE, ensuring AI literacy, and protecting their emotional wellbeing through the rapidly developing environment of generative AI.

## 1 Introduction

Recent advancements in machine learning, particularly the emergence of large language models (LLMs), have marked the beginning of an era dominated by generative artificial intelligence (AI), exerting substantial influence across diverse fields (Miguel and Sarasa-Cabezuelo, 2025), not to mention the specific realms of English as a foreign language (EFL) and second language (L2) instruction. LLMs provide versatile and sophisticated functionalities, fundamentally reshaping traditional pedagogical practices. Leveraging advanced deep-learning architectures, LLMs adeptly recognize intricate linguistic patterns and generate contextually relevant, coherent outputs. Consequently, AI-driven conversational agents, exemplified by platforms such as ChatGPT 4o, DeepSeek, Kimi, and Dou Bao, significantly facilitate personalized language acquisition through authentic communicative interactions, immediate and tailored feedback, and the generation of targeted learning materials aligned with individual needs (Jeon et al., 2024; Wu and Li, 2024).

With the rapid proliferation and increasing accessibility of AI, research in the EFL domain has increasingly focused on AI-mediated informal digital learning of English (AI-IDLE), exploring how learners engage with AI learning tools to foster autonomous English learning beyond formal educational settings (Liu et al., 2024a). In contrast to traditional classroom environments, which can evoke negative emotional experiences such as boredom or anxiety, AI-IDLE enables learners to select personally meaningful and engaging English materials, to explore the pedagogical affordances of AI, including real-time feedback, adaptive correction, and personalized support, and to draw upon its extensive learning resources to enrich their language development. However, the integration of AI into language learning is not exclusively linked to positive outcomes. The use of AI learning tools can also elicit negative emotional responses. Anxiety resulting from AI-assisted learning (AAL), a form of context-specific anxiety that EFL learners experience in AI-facilitated learning environments, intensifies their emotional discomfort and resistance, further damaging their learning performances. Such anxiety may stem from cognitive overload caused by complex intelligent tutoring systems, unfavorable evaluations generated by AI learning tools, challenges in adapting to frequent technological updates, and uncertainty about how to formulate effective prompts (Modliński et al., 2024; Schiavo et al., 2024; Wang et al., 2024). At the same time, research grounded in positive psychology has highlighted that adaptive motivational factors, such as the ideal L2 self (IL2), an envisioned future identity characterized by confident L2 use (Dörnyei, 2009), and positive emotional variables, including foreign language enjoyment (FLE) (Li et al., 2018), can foster EFL learners’ deeper engagement and greater confidence in language learning by enhancing their self-efficacy beliefs (SE) (e.g., An et al., 2021; Wang and Sun, 2024; Hong and Tai, 2025).

Therefore, AAL is expected to be negatively associated with students’ engagement in AI-IDLE. In contrast, a more vivid IL2 is hypothesized to enhance FLE, which in turn promotes learners’ engagement in spoken English practices in AI-IDLE. FLE is also expected to mediate the link between IL2 and SE in speaking ability, such that students who enjoy learning English more strongly feel capable of speaking it, and students’ greater engagement in AI-IDLE is cautiously expected with higher levels of SE in speaking. On this ground, through integrating motivational, psychological, emotional, and extramural factors into a unified model, the present study aims to investigate how Chinese high school students manage AAL while engaging in AI-IDLE, specifically focused on speaking practice. Furthermore, it explores how FLE and IL2 sustain learner engagement in AI-IDLE and contribute to the development of speaking SE, as well as whether AI-IDLE plays a facilitating role in strengthening EFL learners’ SE in spoken English. Lastly, by combining structural equation modeling (SEM) with follow-up qualitative interviews, the study not only tests hypothesized relationships but also contributes to providing a richer, context-sensitive understanding of EFL learners’ experiences with LLMs-based learning tools.

## 2 Literature review

### 2.1 AAL and AI-IDLE

Foreign language classroom anxiety (FLCA) within EFL contexts has received substantial scholarly attention (Dewaele and MacIntyre, 2014), particularly through the theoretical perspective of achievement emotions, wherein the Control-Value Theory (CVT) by Pekrun (2006) has emerged as a notably comprehensive explanatory framework. According to CVT, learners’ emotions primarily arise from two critical cognitive appraisals: The perceived control learners believe they have over learning tasks, and the value they ascribe to these tasks. Within EFL learning environments, anxiety commonly occurs when learners perceive a high importance in achieving language proficiency (high value) yet simultaneously doubt their capability to attain this goal (low perceived control). Such cognitive dissonance is especially prominent in speaking-related tasks and technologically enhanced learning settings, where uncertainty regarding linguistic competence or technological adeptness may intensify learners’ anxiety. In this study, different from FLCA, which generally reflects EFL learners’ negative emotion in the EFL classroom, AAL is conceptualized as a situational state anxiety that arises specifically during interactions with AI learning tools. This AAL is not a permanent trait, but rather an immediate emotional response triggered by AI-mediated learning tasks, for example, cognitive overload or uncertainty when adapting to new AI interfaces, and it aligns with CVT (Pekrun, 2006) when EFL learners perceive low control over an AI-enhanced learning activity despite high value placed on the learning outcome, anxiety is likely to be induced. Recent empirical studies have confirmed the side effects of AAL in AI-blended learning. For instance, Wang et al. (2024), using partial least squares structural equation modeling (PLS-SEM) analysis in the educational environment in Taiwan, found that AAL harmed EFL learners’ attitudes, intrinsic and extrinsic motivational orientation toward language learning. Similarly, a study conducted by Modliński et al. (2024) in Turkey found that struggling to stay current with rapidly evolving AI-facilitated educational tools aroused significant AAL among educators and learners. Additionally, Schiavo et al. (2024) found that AAL functioned in the technology acceptance model (TAM) as a negative mediator, which highlighted the serious role of anxiety in technology-enhanced language learning.

Building upon the extensive body of research on IDLE, including the seminal contributions of Liu and Wang (2024), Zadorozhnyy and Lee (2025), and the thematic synthesis of Liu et al. (2025), the concept of AI-IDLE has been introduced to describe how language learners engage with AI learning technologies to promote autonomous English learning beyond formal educational contexts, and encapsulates diverse informal learning practices undertaken by L2 learners who explore the pedagogical affordances of AI, such as real-time feedback, adaptive correction, and personalized scaffolding, while leveraging its abundant digital resources to experience English across various genres, stylistic registers, and communicative forms. AI-IDLE emphasizes reciprocal interaction with intelligent systems capable of generating customized input, providing adaptive support, and simulating authentic communicative situations. This interactivity highlights the centrality of learner autonomy and agency, as learners actively construct and direct their own language-learning trajectories by negotiating the affordances and limitations of AI learning applications, ranging from basic engagement with AI-generated materials to sophisticated co-creative collaborations with AI systems. These patterns underscore the importance of critical AI literacy, which enables learners to navigate the complex and often opaque power dynamics inherent in AI platforms and to engage in empowered, agentic learning practices. From a psychological standpoint, AI-IDLE is treated as an intrinsically agentic learning process where learners operate as self-regulated, goal-directed participants who proactively seek out and co-construct language resources with AI rather than absorbing content passively under the Proactive Language Learning Theory (PLLT) (Papi and Hiver, 2025). Therefore, AI-IDLE represents a novel intersection between AI-assisted language education and informal learning. While it shares with other forms of AI-supported learning, such as intelligent tutoring systems in formal settings, it is distinct in its learner-driven, interest-oriented, and non-institutional character, operating independently of curricular demands or teacher evaluation.

While prior studies have addressed the relationship between positive emotional constructs, such as enjoyment, and AI-IDLE (e.g., Liu et al., 2024a), to the best of our knowledge, empirical research explicitly examining the impact of negative emotional responses affiliated with AI learning, particularly AAL, on learners’ engagement with AI-IDLE, especially within the context of spoken English skills, remains scarce. Addressing this gap in the literature, the current study proposes the following hypothesis:

*H1*: AAL is significantly related to AI-IDLE.

### 2.2 IL2, FLE, and AI-IDLE

L2 motivation represents a multifaceted construct conceptualized differently across various theoretical frameworks, and the L2 Motivational Self System (L2MSS) proposed by Dörnyei (2009) maintains one of the most influential and widely applied frameworks, especially for operationalizing and assessing motivational orientations among language learners. Central to L2MSS are two distinct self-concept dimensions: IL2 and the ought-to L2 self. Unlike the ought-to L2 self, which captures externally imposed expectations and obligations, IL2 refers to an internally constructed, personally meaningful self-representation that significantly impacts emotional engagement and motivational efficacy (Dörnyei, 2009; Papi et al., 2019). Numerous studies (see review papers in Jin and Lee, 2022) consistently have indicated that learners possessing a vivid and elaborated IL2 demonstrated enhanced persistence, deeper engagement, and superior outcomes in second language acquisition (SLA). Yang and Lian (2023), for instance, found that IL2 significantly predicted learners’ willingness to communicate (WTC), which in turn facilitated pragmatic competence among Chinese EFL university students. Their results revealed a robust pathway from IL2 to communicative outcomes. Moreover, IL2, as an essential motivational construct, has demonstrated robust positive associations with FLE, concurrently framed as an affective state of positive activation that arises when learners experience language tasks as challenging but doable, intrinsically meaningful, and personally rewarding (Li et al., 2018), highlighting the intricate interplay between future-oriented self-guides and learners’ immediate emotional experiences (Lee et al., 2020; Sadoughi and Hejazi, 2024; Wu, 2024). These two categories are highly interrelated: subjects with clear mental images of their IL2 have a predisposition to enjoying learning processes and, consequently, increasing their FLE. These positive emotional experiences then bolster motivational persistence by continually strengthening the learner’s mental representation of her or his idealized future self. Besides, the mediating effects of FLE between learners’ future-oriented self-concepts and informal engagement in technology-enhanced language learning tasks were also validated by the previous literature. For example, Liu et al. (2024b) further suggested that learners with high-level FLE and strong IL2 in parallel were expected to be more active in IDLE. Similarly, learners with a strong IL2 and higher levels of FLE tend to participate more deeply and persistently in AI-mediated informal learning contexts.

While some emerging studies (e.g., Liu et al., 2024a) have focused on IL2 and FLE in the context of AI-IDLE recently, there have been relatively few published studies that specifically explore Chinese EFL high school students’ participation in AI-IDLE to enhance their speaking ability. Liu et al. (2024a) explored the interaction between IDLE practices and the ideal and ought-to L2 selves regarding learner enjoyment through a survey of 690 Chinese university students and a series of qualitative interviews with 12 participants. Their findings reinforced the significant positive relationships between FLE, IL2, and active AI-IDLE participation. Given the established theoretical and empirical links among IL2, FLE, and AI-IDLE from previous studies, the current investigation articulates the following hypothesis:

*H2:* FLE significantly mediates the relationship between IL2 and learners’ engagement with spoken English practices in AI-IDLE.

### 2.3 IL2, FLE, and SE in speaking ability

According to the social cognitive theory (SCT), SE refer to individuals’ judgments about their capabilities to accomplish specific tasks or actions (Bandura, 1997, 2006), and Chacón (2005) further adapted these elements specifically to SLA, operationalizing SE through four fundamental linguistic domains frequently emphasized in the EFL context: listening, speaking, reading, and writing. These beliefs are distinct from broader self-beliefs, such as general self-concept or self-esteem, in that they are task-specific, dynamic, and directly predictive of behavior and achievement. In the context of SLA, SE in speaking pertain to learners’ confidence in their ability to successfully perform oral communication tasks in English.

In addition to the IL2 and FLE connection mentioned before, studies have examined how the IL2 intertwines with SE. Generally, learners who imagine a competent future L2-self also display higher confidence in their current language abilities (Sun and Mu, 2023). For instance, Yang and Lian (2023) found that both IL2 and SE significantly influenced Chinese EFL learners’ performance in L2 pragmatic tasks, with WTC acting as a mediator. The results suggested that when learners see themselves as successful L2 users and believe in their speaking capabilities, they are more likely to engage in communication and thereby perform better, and SE itself can reciprocally boost motivation: as learners gain confidence from successful speaking experiences, their envisioned ideal self grows stronger, creating a positive feedback loop.

Meanwhile, the relationship between FLE and SE has also attracted research interest (e.g., An et al., 2021; Hong and Tai, 2025). FLE can contribute to a learner’s sense of efficacy by reducing anxiety and fostering a safe environment for practice. When students enjoy learning, they are more willing to speak up and take risks, which can improve their speaking skills and reinforce their confidence. Empirical support for this comes from the study of Fathi et al. (2023) on classroom WTC. Positive emotions and SE also interact with each other in the online learning context. For instance, a recent study of Jiang and Yu (2024) found that FLE and grit together predicted learners’ online engagement through the mediation of online learning SE, highlighting that enjoyment contributed to perseverance and confidence in language tasks.

However, it is important to note that SE is domain-specific; learners may have different SE levels for speaking, listening, reading, or writing, and Wang and Sun (2024) have pointed out that research on speaking-specific SE remains relatively scarce. It indicates that while general language SE has been examined extensively, the understanding of learners’ confidence in speaking lags behind. Addressing this gap, we propose the third hypothesis:

*H3*: FLE significantly mediates the association between IL2 and EFL learners’ SE in speaking ability.

### 2.4 AI-IDLE and SE in speaking ability

Although previous studies (e.g., Liu and Wang, 2024) have not conclusively verified the predictive influence of AI-IDLE on SE in speaking, research examining the relationship between IDLE and language learning outcomes has varied. For example, Liu et al. (2025) conducted a thematic review of IDLE research in Asian EFL contexts, noting that IDLE can nurture learners’ WTC and other affective variables. Nonetheless, evidence directly supporting the notion that IDLE participation enhances EFL learners’ learning performances remains ambiguous and inconclusive. Zhang and Liu (2024) reported that engagement in IDLE did not directly predict Chinese university students’ measurable gains in language achievement. Instead, the influence of IDLE on language proficiency was mediated through learners’ cognitive strategy use and motivational regulation. These findings challenge the publicly held assertion that digital English resources inherently lead to huge improvements in language learning outcomes.

While AI-IDLE settings inherently promote learner autonomy, they may fall short in supporting the critical psychological needs of competence, such as offering structured learning tasks with timely, constructive feedback and relatedness, which involves meaningful interactions with peers or instructors. In situations where these psychological needs remain unmet, learners are likely to engage at a surface level without the deep cognitive and emotional commitment necessary for substantial linguistic progress. At the same time, informed by SCT (Bandura, 1997, 2006) and the ecological and sociocultural perspective of van Lier (2004), the quality of AI-IDLE affordances also matters as a boundary condition. When interactions between learners and AI chatbots lack authenticity and social presence, the resulting gains in speaking competence may not effectively translate into enhanced SE (Zadorozhnyy and Lee, 2024). In contrast, more authentic and emotionally engaging exchanges, or hybrid practices that integrate AI-mediated and human communication, are likely to strengthen the positive impact of AI-IDLE on EFL learners’ speaking SE. Considering these theoretical perspectives, we propose the following hypothesis cautiously:

*H4:* AI-IDLE significantly relates to EFL learners’ SE regarding their speaking abilities.

### 2.5 An integrated conceptual framework: self-guided motivation, situational state emotion, AI-IDLE, and SE in speaking

This study is theoretically anchored in an integration of three major frameworks: L2MSS (Dörnyei, 2009), CVT (Pekrun, 2006), and SCT (Bandura, 1997, 2006). Drawing on L2MSS (Dörnyei, 2009), EFL learners’ IL2 is conceptualized as a future-oriented motivational self-guide that shapes both their affective experiences and their engagement behaviors in AI-IDLE. When learners hold a vivid and goal-oriented IL2, they are more likely to perceive AI-based learning activities as meaningful and controllable, which, in turn, fosters higher levels of FLE. In contrast, AAL is treated as a negative situational state emotion that arises during interactions with AI learning tools and can undermine learners’ behavioral engagement in AI-IDLE and weaken their SE in speaking English. From the perspective of CVT (Pekrun, 2006), perceived control operates as a key cognitive appraisal connecting these motivational and emotional processes: a strong IL2 enhances perceived control and supports FLE, whereas AAL signals diminished control over AI-enhanced learning tasks. SCT (Bandura, 1997, 2006) further suggests that these motivational and emotional states reciprocally interact with learners’ behavioral tendencies, such that engagement in AI-IDLE and speaking SE are shaped by the ongoing interplay between personal beliefs and environmental affordances. Taken together, the framework assumes that IL2 exerts both direct and indirect effects on AI-IDLE and SE via FLE, AAL directly hampers AI-IDLE and SE, and AI-IDLE is expected to facilitate speaking SE by providing additional practice opportunities.

**FIGURE 1:**
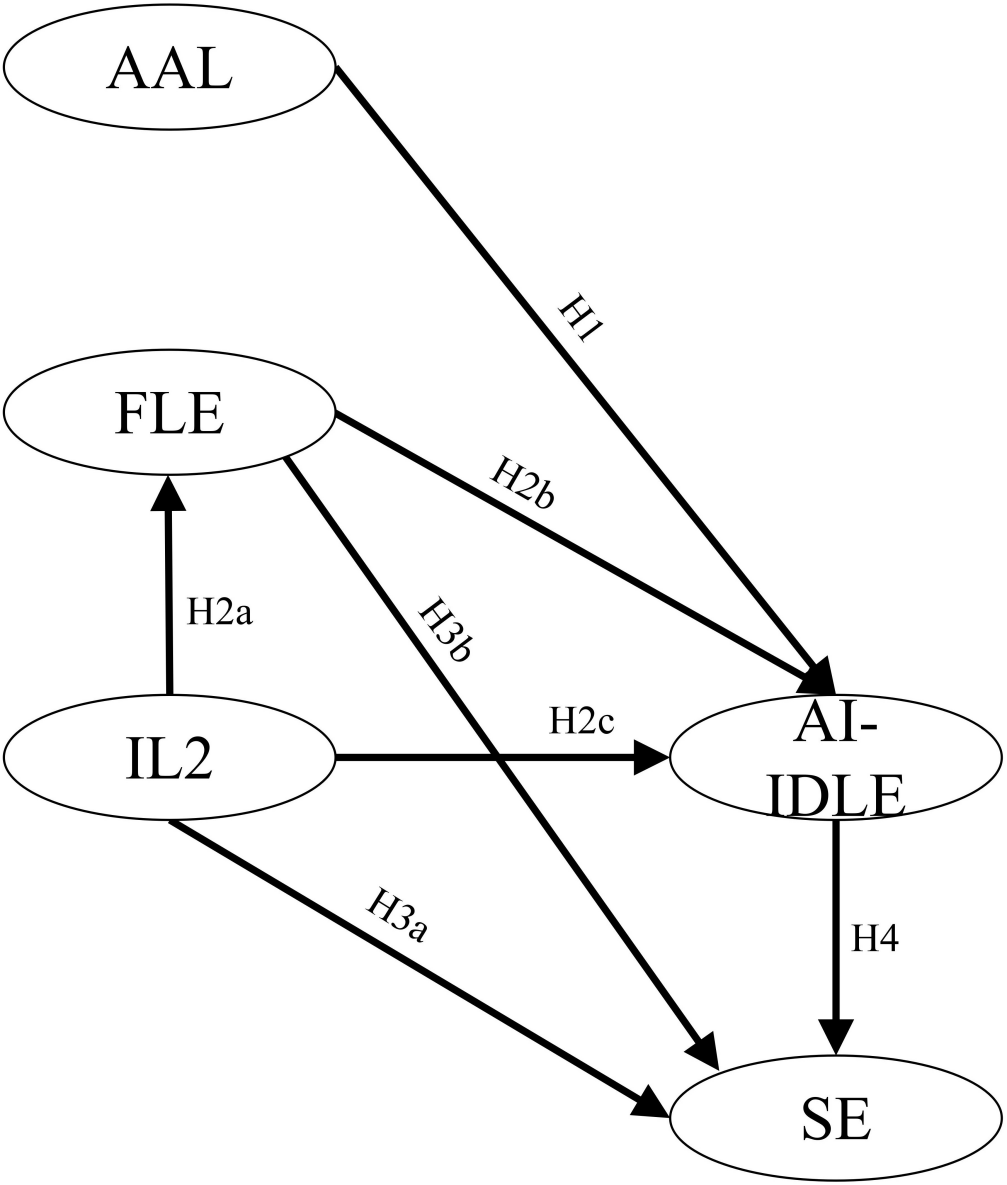
Hypothesized model (H2 consists of H2a, H2b, and H2c; H3 includes H2a, H3a, and H3b).

Although some prior studies (e.g., Liu et al., 2024a) have documented the impact of positive emotion, like FLE, on the relationship between IL2 and AI-IDLE, few have shed light on the less-featured, situational state, and negative emotion like anxiety that arises when EFL learners use AI in learning and investigate the complex relationship in the Chinese high school context, let alone concurrently considered the effect of AI-IDLE on EFL learners’ SE in speaking ability. As presented in Figure 1, this integrated conceptual framework specifies the hypothesized relationships among IL2, FLE, AAL, AI-IDLE, and speaking SE and guides four research questions examined in this study.

*RQ1*: To what extent is AAL associated with EFL learners’ engagement in AI-IDLE, particularly in speaking-related activities?

*RQ2*: In what ways does FLE function as a mediating factor between IL2 and EFL learners’ engagement in AI-IDLE, with a focus on speaking skills?

*RQ3*: How does FLE mediate the relationship between EFL learners’ IL2 and SE concerning English speaking proficiency?

*RQ4*: To what extent is participation in AI-IDLE associated with EFL learners’ SE, particularly in the domain of speaking?

## 3 Materials and methods

This study employed an explanatory sequential mixed-method approach, integrating quantitative and qualitative phases to provide comprehensive insights into the research questions (Mackey and Gass, 2021). Initially, quantitative data were collected and statistically analyzed using a modified questionnaire to examine how constructs such as IL2, AAL, FLE, and AI-IDLE influenced Chinese high school students’ SE in speaking ability. Subsequently, qualitative data were gathered and analyzed based on the preliminary quantitative findings to elucidate deeper motivational and affective nuances that shaped learners’ experiences with AI-IDLE and their perceived speaking competence.

### 3.1 Participants and research design

The quantitative sample consisted of 308 Chinese EFL learners (165 males, 143 females) recruited from four private high schools using simple random sampling. These schools were primarily located in provincial capital cities across mainland China and provided reliable internet access as well as institutional approval for students to use AI-based language learning tools outside regular class hours. Most participants were between 15 and 17 years old (*N* = 260, 84.42%), while a smaller proportion included students older than 17 (*N* = 38, 12.34%) and younger participants under 15 (*N* = 30, 9.74%). The students were evenly distributed across three educational stages: 102 from junior one (33.12%), 124 from junior two (40.26%), and 82 from junior three (26.62%). Over two-thirds (*N* = 248, 80.52%) indicated they owned a smartphone, tablet, or computer with access to AI learning platforms powered by ChatGPT 4o, DeepSeek, Dou Bao, or Kimi, and reported having used such tools for practicing spoken English outside the classroom at least twice a week during the previous month, typically lasting more than 45 min per session.

Additionally, an optional item at the survey’s conclusion assessed participants’ willingness to partake in follow-up qualitative interviews. Consequently, eight interviewees with intermediate English proficiency (B1), as classified by the Common European Framework of Reference for Languages (CEFR) scale (North, 2014), were randomly selected for in-depth qualitative exploration. Interviews were conducted in Chinese to allow participants to express their thoughts and emotions freely. Each interview was audio-recorded with participants’ consent. The recordings were transcribed verbatim in Chinese and then translated into English for reporting in this research. An associate professor with expertise in English-Chinese translation produced the initial translations, which were then checked by another bilingual researcher. Each interview, which lasted between 30 and 60 min, began with a brief review of the participant’s questionnaire responses and then proceeded to an in-depth discussion guided by a semi-structured interview protocol. Example questions included “Do you think using generative AI in your free time can significantly improve your English speaking ability?” and “In what ways do you think your AI-powered informal language learning, motivation, self-efficacy, and enjoyment are connected?” We conducted open and axial coding to analyze the qualitative data following a thematic analysis approach (Dawadi, 2020), with particular attention given to ensuring consistency in the coding outcomes.

### 3.2 Instruments

The study employed a customized 21-item questionnaire comprising five validated scales alongside demographic queries (see Table 1). The questionnaire was administered through *Wenjuanxing*, an online survey platform during regular school hours. After obtaining approval from the university ethics committee, the authors briefly introduced the purpose of the study and shared the survey link with the students. Participation was entirely voluntary, and students could withdraw at any time. To maximize accessibility and reduce comprehension difficulties, the questionnaire was presented in both Chinese and English. The Chinese wording was used as the primary reference for students’ responses, while the English equivalents were provided in parentheses to maintain terminological precision. For the Chinese version, we followed a standard translation and back-translation procedure. First, the items were translated into Chinese by the first author. Second, an independent bilingual expert back-translated the Chinese version into English. Discrepancies between the original and back-translated English versions were discussed and resolved collaboratively. Finally, two associate professors in applied linguistics reviewed the entire questionnaire to ensure conceptual equivalence and clarity. Reliability indices in psychometric analyses (see Table 1) further supported the internal consistency and construct validity of the Chinese version.

**TABLE 1:** Results of exploratory factor analysis.

Items	Factors				
	AAL	IL2	AI-IDLE	SE	FLE
Learning to use specific functions of an AI learning system makes me anxious.	0.930	0.921	0.873	0.872	0.838
Learning to use AI learning systems makes me anxious.	0.922				
Learning how an AI learning system works makes me anxious.	0.922				
Being unable to keep up with the advances in AI learning systems makes me anxious.	0.821				
Taking a class about the development of AI learning systems makes me anxious.	0.761				
I can imagine a situation where I am doing business with foreigners by speaking English.					
I can imagine myself successfully giving a speech in public in English in the future.		0.903			
I can imagine myself talking with foreign friends in English at the party in the future.		0.875			
I can imagine myself chatting easily in English with a foreigner in a cafe with light music playing.		0.846			
I use AI-powered chatbots to practice spoken English with feedback on my fluency and intonation.					
I engage with AI-powered English chatbots on various topics to increase my exposure to English.			0.867		
I play AI-powered language learning games to expand my English vocabulary.			0.853		
I use AI technologies to simulate real-life English language use situations.			0.777		
When interacting with an English speaker, I can participate in a conversation at a normal speed.					
I can express and support my opinions in English when speaking about general topics.				0.836	
I can talk in English about cultural themes and norms in the US.				0.798	
I understand the meaning of common idiomatic expressions when talking with an English speaker.				0.732	
It is fun to learn English.					
I have learned interesting things when learning English.					0.828
There is a positive environment of learning English around me.					0.774
I enjoy learning English.					0.741
Cronbach’s alpha	0.929	0.956	0.919	0.887	0.921

AAL, Anxiety resulting from AI-assisted learning; IL2, Ideal L2 selves; FLE, Foreign language enjoyment; AI-IDLE, AI-mediated informal digital learning of English; SE, Self-efficacy beliefs in English speaking capacity.

The five instruments in the survey employed different Likert-scale ranges of five-point, six-point, and seven-point as we retained each scale’s original response format to preserve its validated psychometric properties. While this may introduce some heterogeneity in response options, we deemed it important to maintain consistency with how each scale was originally developed and validated. We acknowledged this as a methodological consideration; however, for analysis, we treated all scales with appropriate standardization when necessary.

#### 3.2.1 The ideal L2 self-scale

Participants’ IL2 was evaluated using a six-item scale derived from the L2MSS of Dörnyei (2009) and validated in prior studies (e.g., Liu et al., 2024a) conducted in Chinese EFL contexts. Items specifically targeted learners’ envisioned future English-speaking competencies. Responses were scored on a seven-point Likert-type scale ranging from 1 (“totally not like me”) to 7 (“totally like me”), with higher scores indicative of greater intrinsic motivational orientations toward language learning.

#### 3.2.2 The anxiety resulting from AI-assisted learning scale

AAL was measured through an eight-item subscale adapted from the anxiety of learning AI techniques or products dimension of Wang and Wang (2022). This scale explored learners’ apprehensions about acquiring AI-related knowledge, maintaining technological updates, and effectively interacting with AI tools. In adapting the scale, we modified item wording to focus on AI learning tools. For example, an original item, “Learning to use AI systems makes me anxious,” was revised to “Learning to use AI learning systems makes me anxious,” ensuring that each item reflects anxiety stemming from interactions with AI learning tools rather than general technology anxiety. Meanwhile, the adapted items reflected key AI-related stressors identified in the literature, like fear of keeping up with advances of AI learning applications, and uncertainty in interacting with AI learning tutors. This adaptation process was validated by expert review to confirm that the content of each item aligned with the construct of AAL as defined in this study. The learners rated their agreement from 1 (“strongly disagree”) to 5 (“strongly agree”), where higher scores represented elevated anxiety levels experienced in AI-mediated English learning contexts.

#### 3.2.3 The self-efficacy beliefs in speaking ability scale

Learners’ speaking SE was measured using a four-item scale adopted from Chacón (2005). Originally, it held a broader 16-item instrument focusing on EFL learners’ speaking, reading, writing, and listening ability, respectively. This sub-scale specifically addressed students’ beliefs regarding their capability to communicate effectively in English, exemplified by items such as “I can talk about American culture with an English speaker.” The scale utilized a six-point Likert scale ranging from 1 (“strongly disagree”) to 6 (“strongly agree”), with higher scores denoting higher levels of perceived speaking competence.

#### 3.2.4 The foreign language enjoyment scale

Given the diversity of available instruments measuring FLE in applied linguistics research (e.g., Li et al., 2018; Aydın et al., 2024), the selection of an appropriate measure was carefully considered. The study primarily adopted the 11-item scale of Li et al. (2018), specifically tailored to the Chinese high school EFL context. Further, the adoption by Liu et al. (2024a), which assessed Chinese university students’ enjoyment in digital English learning contexts outside formal education, was employed. Consequently, the final scale contained five items capturing students’ levels of enjoyment during English learning, with a response ranging from 1 (“strongly disagree”) to 5 (“strongly agree”). Higher scores reflected greater enjoyment experienced by learners.

#### 3.2.5 The AI-IDLE scale

To examine students’ engagement in AI-IDLE for enhancing their speaking ability, the study adopted the four-item sub-scale targeted on utilizing AI language learning resources, such as chatbots, speech recognition tools, and language learning games to practice English speaking ability in the AI-IDLE scale (e.g., Liu et al., 2024a). Participants rated each item on a six-point Likert scale ranging from 1 (“strongly disagree”) to 6 (“strongly agree”), where higher scores reflected a greater tendency to participate in AI-IDLE speaking activities.

### 3.3 Data analysis

After the questionnaire collection, 5 responses (*N* = 5, representing 1.60% of the total sample) were excluded due to incomplete data or careless responding, resulting in a final set of 308 valid questionnaires for further analysis. Each valid questionnaire was assigned a unique identification code, recorded in Microsoft Excel, and then imported into SPSS 30.0 for subsequent statistical analysis.

Descriptive statistics, such as mean values, frequency distributions, percentages, ranges, and extreme observations, were calculated to assess the overall data distribution and identify any potential outliers. Given that the questionnaire was conceptually based on prior empirical studies, an exploratory factor analysis (EFA) was conducted to clarify the latent factor structure and verify psychometric soundness. Consistent with the methodological recommendations outlined by Hair et al. (2019), the convergent and discriminant validity of the constructs was carefully examined. After confirming all analytical assumptions to ensure methodological rigor, hypothesis testing was carried out using AMOS 29.0 and SPSS 30.0, incorporating bootstrapping techniques to estimate mediation effects as well as direct and total effects. The subsequent section offers a detailed account and interpretation of the empirical results.

## 4 Results

### 4.1 Reliability and validity of the research instruments

Descriptive analysis showed skewness (−0.751 to 0.851) and kurtosis (−0.694 to 0.860) values within acceptable limits, indicating approximate normality. Collinearity diagnostics indicated that the variance inflation factor (VIF) values were below 3.3, suggesting no multicollinearity concerns among the constructs (Hair et al., 2019). Normality checks, consistent with Hair et al. (2019), confirmed the suitability for exploratory factor analysis (EFA). Bartlett’s test of sphericity was significant (χ^2^ = 6169.222, *p* < 0.001), and the Kaiser-Meyer-Olkin (KMO) value (0.898) further supported sampling adequacy. Given that all constructs were measured using a single self-report questionnaire, we examined the potential influence of common method bias (CMB) prior to conducting the main analyses. A Harman’s single-factor test was performed by loading all retained items into an unrotated EFA. The first factor accounted for 39.809% of the total variance, which was below the commonly recommended threshold of 50% (Podsakoff et al., 2003). This result suggested that CMB was unlikely to pose a serious threat to the validity of the findings.

EFA, conducted via principal component factoring (PCF) with varimax rotation, retained factors with eigenvalues above one. Items with loadings below 0.60 or cross-loadings above 0.40 were removed (Hair et al., 2019), leading to the exclusion of AAL items 6−8, IL2 items 5−6, and FLE item 4. We found that removing these items did not undermine the construct coverage of their respective scales; the remaining AAL items still reflected the key aspects of anxiety related to AI learning tools, such as AI language learning tool-use anxiety and AI language learning application-updates anxiety, and the revised FLE scale continued to capture core enjoyment in EFL learning facets. All refined scales maintained strong internal consistency with Cronbach’s α exceeding 0.70, suggesting that the scale modifications preserved reliability and that construct equivalence across scales was largely unaffected by the item reductions. The final solution, yielded five factors explaining 64.063% of total variance, aligning with the instrument’s theoretical structure.

We further employed confirmatory factor analysis (CFA) to validate the measurement model. Convergent validity was supported, as composite reliability (CR) values exceeded 0.70 and average variance extracted (AVE) values surpassed 0.40 (Verhoef et al., 2002). Discriminant validity was also confirmed that the square roots of AVE, reported in Table 2, were greater than the corresponding Pearson correlation coefficients, meeting the criteria outlined by Hair et al. (2019). Discriminant validity was further examined using the heterotrait-monotrait ratio (HTMT). All HTMT values (see Table 3) ranged from 0.090 to 0.619 and were well below the conservative threshold of 0.85 (Henseler et al., 2015). In addition, bias-corrected 95% bootstrap confidence intervals based on 5,000 resamples for each HTMT estimate did not include 1.00. These results provided strong evidence that AAL, FLE, AI-IDLE, IL2, and SE were empirically distinct constructs.

**TABLE 2:** Convergent and discriminant validity of the variables.

Variable	Convergent validity		Discriminant validity					Descriptive statistics		
	CR	AVE	SE	IL2	AI-IDLE	FLE	AAL	Mean	SD	N
SE	0.890	0.670	**0.819**	**0.920**	**0.867**	**0.873**	**0.864**	4.119	1.041	308
IL2	0.957	0.847	0.478**					4.741	1.510	308
AI-IDLE	0.923	0.751	0.362**	0.416**				4.544	0.983	308
FLE	0.927	0.762	0.591**	0.532**	0.551**			3.863	0.794	308
AAL	0.935	0.746	−0.159**	−0.069**	−0.329**	−0.288**		2.592	1.230	308

AAL, Anxiety resulting from AI-assisted learning; IL2, Ideal L2 selves; FLE, Foreign language enjoyment; AI-IDLE, AI-mediated informal digital learning of English; SE, Self-efficacy beliefs in English speaking capacity; the numerals in bold on the diagonals are square roots of the AVE; the inner-construct correlations are shown off diagonally;

** *p* < 0.01 (two-tailed).

**TABLE 3:** HTMT ratios evaluation.

Constructs	AAL	FLE	AI-IDLE	IL2	SE
AAL	0.304 [0.201, 0.405]	0.561 [0.474, 0.639]	0.424 [0.307, 0.534]	0.493 [0.386, 0.589]	
FLE					
AI-IDLE	0.346 [0.239, 0.451]				
IL2	0.090 [0.045, 0.178]	0.547 [0.455, 0.627]			
SE	0.170 [0.083, 0.276]	0.619 [0.521, 0.699]	0.378 [0.255, 0.489]		

AAL, Anxiety resulting from AI-assisted learning; IL2, Ideal L2 selves; FLE, Foreign language enjoyment; AI-IDLE, AI-mediated informal digital learning of English; SE, Self-efficacy beliefs in English speaking capacity; 95% confidence intervals in brackets.

Correlation analysis was performed to explore associations among the key psychological, emotional, and AI-affiliated learning constructs in the study. As shown in Table 2, SE was positively correlated with IL2 (*r* = 0.478, *p* < 0.01), AI-IDLE (*r* = 0.362, *p* < 0.01), and FLE (*r* = 0.591, *p* < 0.01). AI-IDLE was also positively related to FLE (*r* = 0.551, *p* < 0.01). Similarly, IL2 demonstrated positive correlations with AI-IDLE (*r* = 0.416, *p* < 0.01) and FLE (*r* = 0.532, *p* < 0.01), indicating strong interconnections among these positive affective and behavioral factors. In contrast, AAL was negatively correlated with SE (*r* = −0.159, *p* < 0.01), IL2 (*r* = −0.069, *p* < 0.01), AI-IDLE (*r* = −0.329, *p* < 0.01), and FLE (*r* = −0.288, *p* < 0.01), respectively. These results suggested that firmer English-speaking self-efficacy beliefs, stronger visions of ideal L2 selves, greater AI-supported informal learning engagement, and higher enjoyment levels were associated with lower anxiety toward AI in language learning.

To further substantiate the construct validity of the adapted questionnaire, a measurement model encompassing the five latent factors and their corresponding observed items was developed using AMOS 29.0. The adequacy of the model fit was evaluated by examining seven widely recognized fit indices: the chi-square to degrees of freedom ratio (χ^2^/*df*), Comparative Fit Index (CFI), Incremental Fit Index (IFI), Tucker-Lewis Index (TLI), Normed Fit Index (NFI), Root Mean Square Error of Approximation (RMSEA), and Standardized Root Mean Square Residual (SRMR). As summarized in Table 4, the results indicated that the measurement model demonstrated an acceptable fit to the data, with all indices falling within their respective recommended thresholds. Accordingly, these findings provided empirical support for the construct validity of the adapted instrument.

**TABLE 4:** Model fit indices.

Model	χ^2^/*df*	GFI	IFI	TLI	NFI	RMSEA	SRMR
The measurement model	1.968	0.907	0.972	0.967	0.944	0.056	0.045
The structural model	1.845	0.908	0.977	0.973	0.952	0.052	0.065
Recommended value	≤3.000	≥0.900	≥ 0.900	≥0.900	≥ 0.900	≤0.080	≤ 0.080

### 4.2 Quantitative results related to the RQs

#### 4.2.1 RQ1: influence of AAL on AI-IDLE

AI-mediated informal digital English learning and anxiety regarding AI-assisted learning were found to be significantly negatively correlated, as shown in Table 5, with AAL emerging as a significant negative predictor (β = −0.240, *p* < 0.001, *t*-value = −4.421). This result empirically supported Hypothesis 1 (H1), which stated that EFL learners’ participation in informal, AI-mediated English learning activities, especially speaking-related ones, may be hampered by higher anxiety levels linked to the use of AI in learning contexts.

**TABLE 5:** Hypothesis test results.

Hypotheses and path relationships	β	*p*	*T*-value	Results
H1: AAL → AI-IDLE	−0.240***	*p* < 0.001	−4.421	Accept
H2a: IL2 → FLE	0.526***	*p <* 0.001	9.999	Accept
H2b: FLE → AI-IDLE	0.384***	*p* < 0.001	6.462	Accept
H2c: IL2 → AI-IDLE	0.199***	*p* < 0.001	3.372	Accept
H3a: IL2 → SE	0.159**	*p* < 0.01	2.658	Accept
H3b: FLE → SE	0.455***	*p* < 0.001	6.740	Accept
H4: AI-IDLE → SE	0.105	0.078	1.764	Reject

AAL, Anxiety resulting from AI-assisted learning; IL2, Ideal L2 selves; FLE, Foreign language enjoyment; AI-IDLE, AI-mediated informal digital learning of English; SE, Self-efficacy beliefs in English speaking capacity;

***p < 0.001;

**p < 0.01.

#### 4.2.2 RQ2: mediating function of FLE between IL2 and AI-IDLE

**FIGURE 2:**
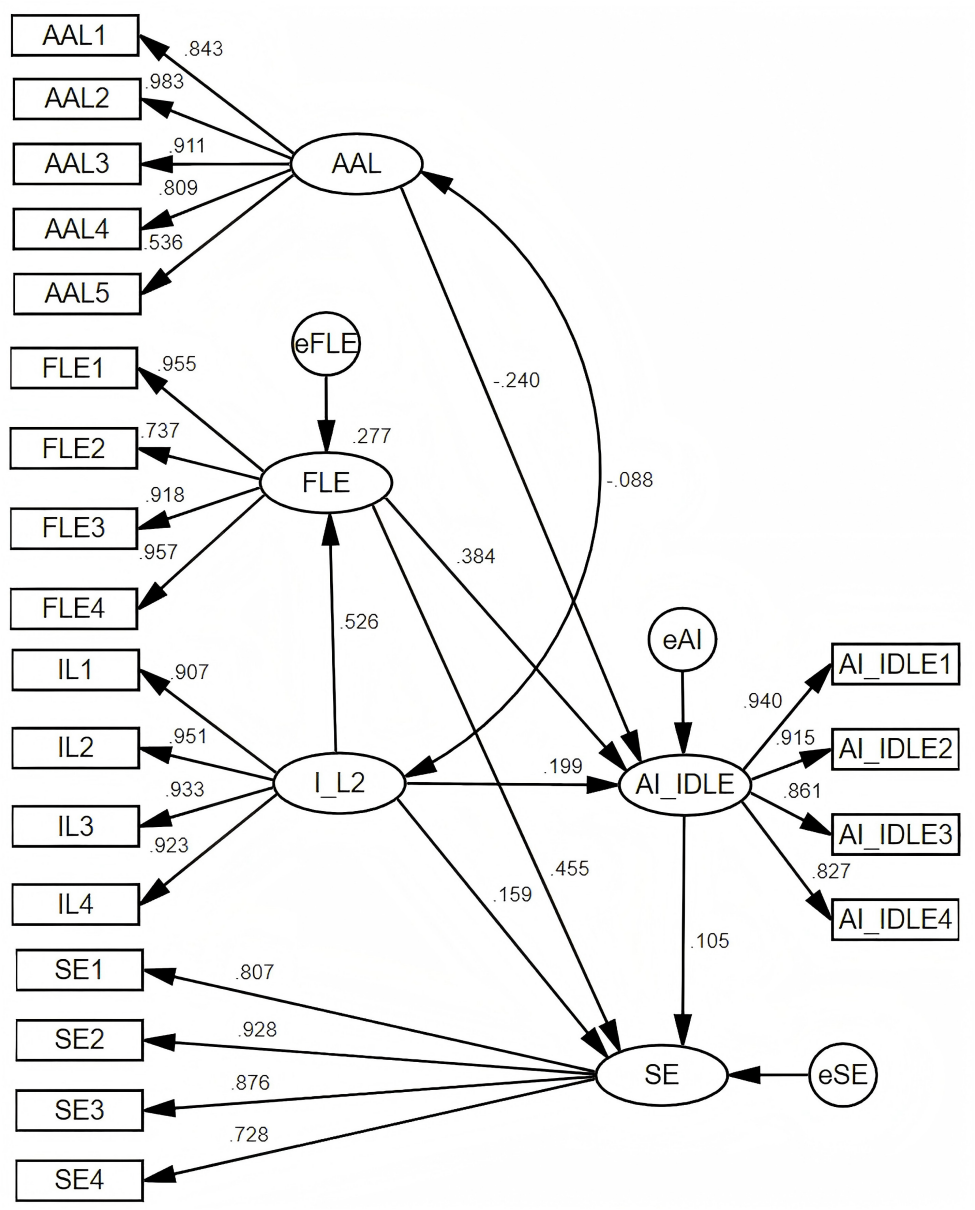
Parameter estimates of the structural model (R^2^: FLE = 27.7%, AI-IDLE = 34.3%, SE = 38.2%).

The mediating mechanism involving FLE was evaluated to test H2a, H2b, and H2c. Table 5 presented the results showing that the path from IL2 to FLE (H2c: β = 0.526, *p* < 0.001, *t* = 9.999), FLE to AI-IDLE (H2b: β = 0.384, *p* < 0.001, *t* = 6.462), and from IL2 to AI-IDLE (H2a: β = 0.199, *p* < 0.001, *t* = 3.372) were statistically significant. Looking at the *R*^2^ in Figure 2, it was found that IL2 accounted for 27.7% of the variance in FLE, while the three variables, AAL, FLE, and IL2, could work together to explain 34.3% of the total change in AI-IDLE. It should be noted that the model’s explanatory power was moderate, though still substantial (Hair et al., 2019). The *R*^2^ values indicated that approximately one-third of the variance in the outcome variables was accounted for, suggesting the presence of other influential factors beyond the current model. While the quantitative findings offered valuable insights, they did not capture the full complexity of the phenomenon, underscoring the need to complement these findings with qualitative evidence.

Given that there existed a potential mediating variable, FLE, which linked IL2 and AI-IDLE, we conducted a mediation test to explore the interplay of IL2, FLE, and AI-IDLE. A bootstrapped analysis with 2,000 samples and a 95% confidence interval was thus undertaken in AMOS 29.0. As shown in Table 6, it was revealed that FLE could partially mediate the impact of IL2 on AI-IDLE (lower bound = 0.096, upper bound = 0.213) as the upper and lower bounds of the mediation path were above zero, and accounted for 60.1% of the total effect in this IL2 → FLE → AI-IDLE mediation model.

**TABLE 6:** Direct and indirect path coefficients of the structural model.

Effect	Path relationship	Point estimate	Product of coefficients		Bootstrapping 95% CI	
					Bias-corrected	
			*SE*	*T*-value	Lower	Upper
IE1	IL2 → FLE → SE	0.146	0.029	5.034	0.096	0.213
IE2	IL2 → FLE → AI-IDLE	0.139	0.029	4.793	0.091	0.210
DE1	IL2 → SE	0.097	0.043	2.256	0.012	0.184
DE2	IL2 → AI-IDLE	0.137	0.051	2.686	0.034	0.240
TE1	IE1 + DE1	0.242	0.047	5.149	0.163	0.353
TE2	IE2 + DE2	0.275	0.051	5.392	0.173	0.381
Contrast 1	IE1 vs. TE1	0.601	0.134	4.485	0.383	0.930
Contrast 2	IE2 vs. TE2	0.504	0.129	3.907	0.302	0.829
Contrast 3	DE1 vs. TE1	0.399	0.134	2.978	0.070	0.617
Contrast 4	DE2 vs. TE2	0.496	0.129	3.845	0.171	0.698

IE, Indirect effect; DE, Direct effect; TE, Total effect; AAL, Anxiety resulting from AI-assisted learning; IL2, Ideal L2 selves; FLE, Foreign language enjoyment; AI-IDLE, AI-mediated informal digital learning of English; SE, Self-efficacy beliefs in English speaking capacity.

#### 4.2.3 RQ3: mediating function of FLE between IL2 and SE

Another mediating mechanism involving FLE was evaluated to test H3a and H3b. Table 5 presented the hypothesis testing results demonstrating that paths from FLE to SE (H3b: β = 0.455, *p* < 0.001, *t* = 6.740) and IL2 to SE (H3a: β = 0.159, *p* < 0.01, *t* = 2.658) were statistically significant. Looking at the *R*^2^ in Figure 2, it was found that variables IL2 and FLE could work together to explain 38.2% of the total change in SE, which reflected at least moderate to substantial explanatory power (Hair et al., 2019). Therefore, the observed *R*^2^ values exceeding 0.30 in this study supported the presence of meaningful, practically significant relationships.

In addition, the indirect effect of IL2 on SE via FLE indicated in Table 6 was statistically significant (95% CI ranging from 0.091 to 0.210), suggesting the presence of a mediating mechanism and indicating that FLE partially mediated this association and accounted for 50.4% of the total effect.

#### 4.2.4 RQ4: influence of AI-IDLE on SE

We further investigated the potential influence of EFL learners’ engagement in AI-mediated informal digital English learning on their self-efficacy beliefs related to English speaking proficiency. This inquiry aimed to explore whether learners’ informal interactions with AI tools, outside the constraints of formal classroom settings, contributed meaningfully to their confidence in spoken language performance.

However, as presented in Table 5, the structural path from AI-IDLE to SE was found to be statistically non-significant (H4: β = 0.105, *p* = 0.078, *t* = 1.764). Although the direction of the relationship was positive, the effect did not reach the conventional threshold for statistical significance (*p* < 0.05), which meant H4 was not tested. This suggested that, in the current model, AI-IDLE alone may not be a reliable predictor of EFL students’ self-efficacy in speaking English. Possible explanations for this non-significant finding were discussed in the following section.

### 4.3 Qualitative results

Complementing the quantitative results, qualitative data from detailed interviews with eight participants provided rich, varied insights into how AAL, IL2, FLE, and SE in speaking ability dynamically interacted and shaped learners’ engagement with AI-IDLE. The interviewees’ nuanced narratives not only corroborated the quantitative findings but also offered profound contextual understandings. The following parts mainly discussed the negative influence of AAL on AI-IDLE and the limited effect of engagement in AI-IDLE on EFL learners’ SE in speaking ability.

The qualitative data vividly illustrated the significant negative impact of AAL on learners’ participation in AI-IDLE activities, as identified in the quantitative results. Participants consistently highlighted experiences of cognitive overload, fear of negative evaluation, difficulties adapting to rapid technological changes, and uncertainty about effective prompt creation, significantly heightening their emotional resistance. Lina (15, Junior 1) described her experiences in detail, emphasizing the cognitive overload and subsequent emotional distress:

*When I first started using a spoken English practicing AI agent, I was genuinely excited. However, as the conversation continued, it began giving me lengthy, complicated responses very quickly. Each time this happened, my mind would freeze. I couldn’t process all the information fast enough, and I felt overwhelmed by its frequently updated interface. I would doubt my ability to understand or respond effectively, and this anxiety kept building.*

Lina’s experience illustrated how excessive cognitive load undermined her perceived control over the task, directly triggering heightened anxiety, which was aligned with CVT (Pekrun, 2006), as low perceived control combined with high subjective value was a formula for anxiety. She highly valued the speaking practice, a priority driven in part by her IL2 under L2MSS (Dörnyei, 2009), but felt unable to manage the torrent of AI learning tools-provided content, then experienced intense negative emotion. Lina valued the activities in AI-IDLE as important for her progress, yet the AI learning applications’ rapid, unfiltered feedback meant she lost control over her learning pace. This imbalanced high value but low control explained why her anxiety spiked. In other words, the deluge of information from the AI tutor eroded her sense of control and thus provoked the very nervousness that stifled her speaking. Therefore, an AI learning tool’s affordances should be carefully calibrated to the learner’s capacity; otherwise, the tool’s power of containing abundant information can backfire by overwhelming the learner and undermining their confidence. Lina’s account powerfully confirmed CVT (Pekrun, 2006) behind AAL: when a learner’s desire to improve, rooted in a vivid IL2, was thwarted by feeling unable to cope, the result was heightened anxiety and loss of motivation to continue.

Despite AI-IDLE’s potential, qualitative data helped explain why quantitative analysis did not find a significant improvement in learners’ speaking SE. Participants provided detailed explanations, pinpointing specific limitations of the current AI-powered speaking tool in IDLE. Michael (16, Junior 2) discussed the artificial nature of AI interactions:

*When I started using ChatGPT’s voice chat, I hoped it would dramatically boost my speaking confidence. Initially, the experience seemed helpful, but soon I realized conversations were too formal, rigid, and lacked emotional depth. Interactions with my foreign tutor, Sam, after school were unpredictable and emotionally charged, very different from the structured AI dialogues. This discrepancy made real-life speaking even more daunting, limiting any transferable gains in speaking confidence from AI practice.*

This comment underscored that while AI chatbots can simulate dialogue, they failed to provide genuine social presence or emotional resonance. Michael’s difficulty suggested that authenticity and interpersonal connection were key for building speaking self-efficacy, which were elements of the learning experience that current AI tools struggled to offer. Moreover, Michael’s experience also warned that over-reliance on AI chats for practice can negatively impact real human connections and individual confidence, meaning gains with a bot might not transfer to real-life situations. The AI-IDLE environment did support his autonomy, as he could practice anytime, free from judgment, but it did not satisfy his need for relatedness, the sense of genuine human connection, which was a basic need for sustaining motivation and confidence (Dörnyei, 2009). Without the authentic social context that imbued conversation with unpredictability, emotion, and mutual presence, Michael found that his improvements with the AI felt hollow. Adrian (17, Junior 3) detailed issues with repetitive and unrealistic content in the Call Annie app, which he used for preparing the speaking part in the International English Language Testing System (IELTS) test:

*At first, I liked the daily speaking tasks provided by Call Annie. However, they quickly became repetitive, and the conversations rarely reflected real-life scenarios or practical vocabulary I needed in the IELTS spoken test. Soon, the tasks felt artificial and disconnected from actual contexts. Ultimately, my motivation dropped significantly, and I questioned whether these exercises genuinely enhanced my practical speaking abilities.*

Adrian provided further insight by focusing on content relevance. His critique of the AI learning tool’s repetitiveness and artificiality illustrated how disengagement can result from a mismatch between learners’ needs, like IELTS test preparation, and the AI content offered. His declining motivation and skepticism about the app’s value reflected a breakdown in the belief that AI-IDLE was a meaningful learning context. This misalignment between learner goals and AI tool design helped explain the lack of significant gains in SE observed in the quantitative analysis. Overall, these detailed qualitative insights illuminated the gap between what AI-IDLE currently offered and what learners required for authentic and confidence-building speaking experiences, revealing a tension between technological affordances and affective authenticity in AI-mediated learning. On one hand, AI learning tools afforded unprecedented opportunities for practice and feedback at any time; on the other hand, if those opportunities overwhelmed learners or lacked human-like authenticity, the emotional and motivational payoffs diminished. This tension emphasized that while AI-IDLE can offer practice opportunities, its impact on SE remained constrained unless the learning context approximated the social and emotional realities of actual communication.

## 5 Discussion

Addressing the first research question, the study confirmed that AAL significantly and negatively predicted EFL learners’ AI-IDLE. This result aligned with CVT (Pekrun, 2006), which posited that high anxiety arose from low perceived control over important tasks. In the AI-IDLE context, students who felt overwhelmed by unfamiliar AI tools with low control showed reduced effort in AI-IDLE, even if they valued improving their English. This finding also echoed the existing literature highlighting the debilitating effect of anxiety on learners’ engagement with technology-enhanced language learning environments (Modliński et al., 2024; Schiavo et al., 2024; Wang et al., 2024). Qualitative data further illuminated this negative impact, vividly captured in participants’ narratives. The interviewee’s experience of cognitive overload and emotional distress underscored how excessive informational demands from AI learning tools triggered anxiety, significantly reducing her motivation and participation, and her struggle with rapid technological changes and difficulties in creating effective prompts revealed that anxiety went beyond linguistic competence to include technological self-efficacy. Simultaneously, this dynamics extended SCT (Bandura, 1997, 2006) by showing that technological self-efficacy in handling the AI learning resources was an important facet: as students became more familiar with the AI learning platform and learned to interpret its feedback, their anxiety subsided, and engagement with AI-IDLE recovered. These insights confirmed that learners’ anxiety within AI-assisted learning contexts was multidimensional, encompassing cognitive, emotional, and technical aspects that collectively influenced their engagement.

Addressing the second research question, the results confirmed that FLE played a mediating role between IL2 and EFL learners’ engagement in AI-IDLE. In line with L2MSS (Dörnyei, 2009), students with a vivid, personally meaningful vision of their future English-using self-exhibited higher enjoyment during AI-mediated informal English learning, which in turn fueled sustained use of AI learning kits. Our findings converged with prior evidence that linked IL2 and FLE to sustained language learning behaviors (e.g., Fathi et al., 2023; Hong and Tai, 2025), while extending this pattern to the emerging context of AI-assisted informal learning (e.g., Liu et al., 2024b) by demonstrating this link in an AI-assisted context where design features like interactive chatbots, immediate feedback, and gamified challenges of AI learning platforms likely contributed to enjoyment, thereby encouraging continued engagement. This mechanism was consistent with recent studies of Liu et al. (2024a), showing that enjoyable interactions can sustain motivation in AI-IDLE. The interview narratives enriched this picture by underscoring how IL2 inspired and guided students’ informal learning. One student visualized herself as a future English tour guide and was intrinsically motivated to practice speaking with generative AI tools daily to approach her dream. She described a genuine enjoyment in celebrating small wins like mastering a new phrase or accent in the learning platform, which kept her coming back to the application. Another learner aimed to speak English with a British accent; his clear ideal self led him to treat the AI’s pronunciation feedback as a fun challenge rather than a chore. These cases illustrated how a well-developed IL2 can spark enjoyment even in self-directed AI practice, as IL2 provided personal relevance and meaning to the task, turning some repetitive chatbot drills into engaging steps toward a valued goal.

The third research question explored the mediating effect of enjoyment between ideal L2 self and self-efficacy beliefs in speaking, supported by both a significant indirect relationship of IL2 → FLE → SE and a direct relationship between IL2 and SE. The observed moderate-to-substantial explanatory power corroborated existing studies emphasizing enjoyment as a pivotal emotional factor enhancing learners’ perceived language competence. This finding resonated with the arguments of previous studies (e.g., Fathi et al., 2023; Sun and Mu, 2023; Jiang and Yu, 2024) on the reciprocal relationship between positive emotional experiences and self-efficacy beliefs. The qualitative narratives from participants also reinforced this point, demonstrating how enjoyable experiences with AI interactions strengthened learners’ belief in their communicative competence as well as potentially illustrated the dynamic and bidirectional relationships among enjoyment, motivation, and self-efficacy. One interviewee described how vividly picturing herself holding conversations made AI-assisted speaking tasks feel meaningful and enjoyable; this enjoyment, in turn, emboldened her to take risks, strengthened her belief that she could succeed in real-life speaking scenarios, and motivated her to return to the AI chatbot more frequently. Another explained that what began as purely task-oriented use of the AI, like completing speaking drills for practice, gradually became intrinsically rewarding: as he noticed small improvements and experienced more enjoyable interactions, his confidence in his speaking ability increased, which heightened confidence, then fed back into a greater willingness to experiment with new topics and more demanding AI-facilitated conversations. Therefore, these narratives not only corroborated the quantitative mediation pattern whereby IL2 enhanced FLE, which in turn facilitated SE in speaking, but also pointed to the possibility of more reciprocal relations among these three constructs in a mutually reinforcing system. This configuration resonated with SCT (Bandura, 1997, 2006), which posited dynamic interplay between motivation, affect, and behavior. Personal factors such as enjoyment, self-efficacy beliefs, and behavioral engagement in speaking practice continuously shaped one another. Thus, the mixed-methods evidence in this study supported viewing L2 motivation and emotion as a dynamic system rather than a strictly linear sequence.

In regard to the fourth question, unexpectedly, the direct influence of AI-IDLE on learners’ self-efficacy beliefs in speaking did not reach statistical significance. This non-significant association diverged from findings in earlier research on traditional technology-assisted informal language learning, where frequent computer-mediated practice has been shown to enhance learners’ WTC (see review papers in Liu et al., 2025). However, it aligned with the large-scale research by Zhang and Liu (2024), emphasizing the authentic L2 experience in the IDLE context. This discrepancy suggested that not all forms of digital practice provided the kind of mastery experiences to strengthen self-efficacy beliefs, especially when interactions lack authentic social and emotional cues. In other words, merely spending time on AI-mediated informal spoken English practice did not automatically translate into greater confidence in speaking ability. This non-finding invited critical reflection, as it initially seemed to contradict expectations from SCT that mastery practice should build self-efficacy (Bandura, 1997). Nevertheless, not all linguistic practices in AI-IDLE were equal in providing mastery experiences. The qualitative interviews further shed light on this: EFL students pointed out that AI-driven speaking practice often lacked authentic social and emotional cues, making it less effective in bolstering their real-world confidence. Several participants noted that while AI chatbots helped them polish grammar or pronunciation, the interactions felt artificial or scripted. This gap between digital rehearsal and genuine communication can limit gains in self-efficacy, as true language proficiency and confidence were developed through socially authentic interaction (van Lier, 2004). It further resonated with the findings of Zadorozhnyy and Lee (2024) that AI-mediated informal learning environments may support EFL learners’ self-efficacy beliefs to some extent, but such beliefs alone were insufficient to drive substantial improvements in autonomous language learning behaviors. Thus, if AI learning platforms failed to simulate the unpredictability and emotional investment of real dialogue, EFL learners may not develop a robust belief in speaking English fluently in real situations.

## 6 Implications

This study explored how Chinese high school students’ anxiety in AI learning, ideal L2 self, and foreign language enjoyment interacted with AI learning tools for the informal learning of English and their influence on students’ self-efficacy beliefs in speaking ability. Some theoretical, methodological, and practical implications can be addressed as follows.

### 6.1 Theoretical and methodological implications

Theoretically, this study provided implications of motivation and emotion in AI-mediated informal English learning in several ways. First, by integrating SCT (Bandura, 1997, 2006), L2MSS (Dörnyei, 2009), and CVT (Pekrun, 2006) as guiding frameworks, the study demonstrated how these perspectives complemented one another within the context of AI-IDLE. Specifically, it extended SCT (Bandura, 1997, 2006) into the domain of generative AI language learning, confirming that traditional constructs such as self-efficacy beliefs and anxiety remained central even when learning occurred autonomously and was supported by advanced AI technologies. Second, the study contributed to L2MSS (Dörnyei, 2009) by revealing that the ideal L2 self-continued to drive learner engagement and positive emotions beyond formal instructional contexts. It also provided empirical evidence that a vivid and future-oriented L2 self-enhanced foreign language enjoyment, which in turn fostered persistence in using AI-assisted learning tools. These findings underscored the motivational force of future self-guides in sustaining engagement within emerging AI-mediated informal learning environments such as AI-IDLE. Third, the study enriched the application of CVT (Pekrun, 2006) in SLA by clarifying how EFL learners’ anxiety in AI-facilitated learning operated as a negative situational emotion that diminished their engagement in AI-IDLE. While prior studies have associated general anxiety with various learning outcomes, the present research identified the specific pathways through which situational state anxiety in AI-facilitated language learning constrained learner participation.

Methodologically, this research demonstrated the value of an explanatory sequential mixed-methods design in educational psychology. By complementing SEM with in-depth interviews, this research was able to validate, interpret, and uncover the quantitative patterns with real-world context, as well as why certain effects and non-effects occurred from qualitative insights. Such rich, triangulated evidence was a notable contribution to the field, as studies in the AI-based informal learning field were often solely quantitative. The explanatory mixed-methods strategy can serve as a model for future investigations aiming to capture both breadth and depth, as surveys can map broad relationships among new constructs, like AI-IDLE and AAL, and interviews can reveal learners’ subjective experiences and evolving perceptions that underpinned those relationships. Additionally, the adapted, validated, and contextualized measurement instruments like the AAL scale for AI-facilitated learning can be used or further refined in subsequent studies, helping to build a cumulative research base in this nascent area.

### 6.2 Practical implications

The findings also offered several actionable insights for EFL educators, learners, and AI tool designers aiming to support informal digital English learning. First, simply providing AI learning platforms to students is not a magic bullet for improving language skills, as EFL learners’ emotional and motivational readiness must be addressed. For instance, to mitigate anxiety resulting from using AI learning resources, educators could offer low-stakes and hands-on sessions that build AI literacy, confidence, and familiarity in manipulating these tools, which include guided practice with AI learning resources in a supportive environment so that students may feel more in control, thereby reducing anxiety at the outset. Second, AI learning tools could include interactive and gamified features that sustain motivation, such as adaptive feedback, progress badges, or immersive simulations. Teachers can reinforce this by incorporating reflective tasks that help students connect enjoyable experiences with their learning goals. Moreover, learners should be encouraged to visualize their ideal L2 self through practical, goal-setting tasks. Activities such as digital vision boards or personal language portfolios can help solidify these imagined identities.

Teachers might also organize regular check-ins to discuss students’ progress toward these visions, thereby maintaining motivational momentum. Given the limited effect of AI-IDLE on speaking self-efficacy beliefs, improving the authenticity of AI interactions is critical. AI learning platforms with less scripted, more emotionally responsive AI dialogues will be more welcome. Culturally relevant, real-life scenarios can also bridge the gap between digital rehearsal and authentic communication, and a blended model, combining AI tools with peer or teacher-led speaking tasks, may offer a more emotionally resonant and skill-building experience. Lastly, learners should be trained to assess and choose AI tools that align with their communicative goals and language proficiency. Instructors in the learning community can offer short workshops or guides to help students critically evaluate AI platforms based on relevance, interactivity, and effectiveness. Empowering learners with evaluative strategies will strengthen their autonomy and long-term self-efficacy in digital language learning.

## 7 Conclusion

This study investigated how Chinese high school students’ AAL, IL2, and FLE interacted with AI-IDLE and SE in speaking ability. Using an explanatory sequential mixed-methods design, this research found that AAL operated as a detrimental, situation-specific achievement emotion that undermined engagement in AI-IDLE, whereas a vivid IL2 and higher levels of FLE supported more sustained participation in speaking-oriented learning activities in the AI-IDLE context. FLE emerged as a key affective conduit through which IL2 translated into stronger beliefs about speaking ability, highlighting the importance of positive emotions in turning future self-guides into perceived competence. At the same time, the non-significant direct path from AI-IDLE to speaking self-efficacy, together with the interview, accounted for cognitive overload and emotionally flat in AI dialogues, suggesting that not all AI-based practice readily transferred into confidence for real-life communication.

Overall, these findings indicated that the pedagogical promise of AI-IDLE for adolescent EFL learners did not lie in increasing digital practice alone, but in orchestrating AI use within a broader motivational and emotional ecology. When AI-mediated activities were aligned with learners’ IL2, infused with FLE rather than AAL, and embedded in supportive human-AI informal learning environments, they were more likely to contribute meaningfully to the development of speaking self-efficacy beliefs. By foregrounding the intertwined roles of IL2, FLE, AAL, and AI-IDLE, the present study offered an empirically grounded starting point for designing emotionally sustainable, theoretically informed uses of generative AI in secondary EFL education.

## 8 Limitations and future directions

While this study provided valuable insights into the affective and motivational dynamics of AI-mediated informal digital English learning, several limitations should be acknowledged. First, the sample size and demographic composition were limited to Chinese senior high school students, which may affect the generalizability of the findings across broader cultural or educational contexts. Second, the qualitative phase involved only several participants, which, while providing rich contextual data, may not capture the full diversity of learner experiences, and the fast-evolving nature of AI tools means that learners’ experiences may change rapidly, potentially rendering some aspects of the findings quickly outdated. Lastly, the cross-sectional design limited our ability to infer temporal or causal relationships, future research should expand participant diversity and explore longitudinal patterns to further enrich the understanding of AI-IDLE.

## Data Availability

The raw data supporting the conclusions of this article will be made available by the corresponding author upon reasonable request.
